# Role of Insulin-Like Growth Factor Receptor 2 across Muscle Homeostasis: Implications for Treating Muscular Dystrophy

**DOI:** 10.3390/cells9020441

**Published:** 2020-02-14

**Authors:** Yvan Torrente, Pamela Bella, Luana Tripodi, Chiara Villa, Andrea Farini

**Affiliations:** Stem Cell Laboratory, Department of Pathophysiology and Transplantation, University of Milan, Unit of Neurology, Fondazione IRCCS Cà Granda Ospedale Maggiore Policlinico, Dino Ferrari Center, 20122 Milan, Italy; pamelabella@hotmail.it (P.B.); tripodiluana@libero.it (L.T.); kiaravilla@gmail.com (C.V.)

**Keywords:** IGF2R, muscle homeostasis, inflammation, muscular dystrophy, pericytes

## Abstract

The insulin-like growth factor 2 receptor (IGF2R) plays a major role in binding and regulating the circulating and tissue levels of the mitogenic peptide insulin-like growth factor 2 (IGF2). IGF2/IGF2R interaction influences cell growth, survival, and migration in normal tissue development, and the deregulation of IGF2R expression has been associated with growth-related disease and cancer. IGF2R overexpression has been implicated in heart and muscle disease progression. Recent research findings suggest novel approaches to target IGF2R action. This review highlights recent advances in the understanding of the IGF2R structure and pathways related to muscle homeostasis.

## 1. Introduction

The cation-independent mannose 6-phosphate/insulin-like growth factor 2 receptor (CI-M6P/IGF2R, hereafter IGF2R) is a type-1 transmembrane glycoprotein consisting of a large N-terminal extracytoplasmic domain, which allows it to bind to a wide variety of ligands [1,2]. The IGF2 and M6P ligands [3,4,5] of IGF2R have distinct but important roles in normal development and mesoderm differentiation [6]. Many studies have demonstrated the suppression action of IGF2R on insulin-like growth factor 1 receptor (IGF1R) signaling by scavenging extracellular IGF2 [7]. Furthermore, several lines of evidence demonstrate that IGF2 is highly expressed in rodent embryos, where it functions as an embryonic growth factor, while its amount is diminished at birth [8]. Smith et al. recently showed that a transgene-induced overexpression of IGF2 blocked programmed cell death, one of the main pathological features of cancer [9]. Furthermore, in some cancers such as mammary tumors, IGF2R behaves as a tumor suppressor gene [10], whereas in other cancers such as cervical tumors or glioblastomas, IGF2R acts as an oncogene [11,12]. Thus, these two traits of IGF2R might depend on cell type. Interestingly, cervical tumors and glioblastomas have common mesenchymal founders, namely myofibroblasts [13], which are also involved in muscle disease. Muscle repair is a complex and tightly regulated event that recruits different cell types, starting from macrophage and lymphocyte consecutive involvement and terminating with satellite cell (SC) activation and differentiation [14]. Among the common hallmarks of muscular dystrophy are the infiltration of immune cells into skeletal muscle fibers, and fibrotic cell proliferation [15,16,17,18].

Impaired muscle regeneration with SC pool exhaustion is considered an additional pathological feature of Duchenne muscular dystrophy (DMD) [19]. The main biological function of IGF2R is the suppression of IGF1R signaling via the deprivation of extracellular IGF2 ligands. Some studies have explained the tumor suppressive functions of IGF2R by its negative regulation of the oncogenic IGF2–IGF1R signal axis [2]. However, in muscle tissues, the IGFs bind to the IGF1R leading to conformational changes and activation of its tyrosine kinase activity, promoting muscle regeneration [20]. In injured tissues, IGF1 is secreted by SCs and mediates muscle-derived stem cell proliferation and differentiation into myoblasts, which contribute to myofiber formation for restoring normal tissue structures [21]. It has been demonstrated that prolonged expression of IGF1 causes an exaggerated protein synthesis and is responsible for muscular hypertrophy, by increasing myofiber diameter [22,23,24,25], and also preventing muscle atrophy in cases of cachexia or chronic inflammation [26]. Thus, tissue-specific IGF1 upregulation rises to the challenge of counteracting the development of muscular dystrophy.

Similar to IGF1, autocrine IGF2 is fundamental to mediate the differentiation of SCs in vitro, but little is known about its role in skeletal muscle development and regeneration in vivo [27]. The expression profile of *IGF2* is quite complicated as it depends on multiple-promoter activation, alternative translation initiation and messenger RNA (mRNA) stability. IGF2R functions as a negative regulator of IGF2 in embryonic skeletal muscles and modulates the amount of systemic IGF2 by inducing its degradation through lysosomes and clearance from the circulation [28,29].

Even if the signaling cascade that regulates the activation of IGF2 at muscular level is not determined, it has been shown that the phosphatidylinositol 3-kinase (PI3K)–the serine/threonine protein kinase B (AKT) pathway is the IGF2 downstream pathway contributing to mammalian target of rapamycin (mTOR) functions [30]. A study by Ge et al. [31] showed that the synergic activity of mTOR with miR-125b regulated *IGF2* production both transcriptionally and post-transcriptionally, and that these events positively influence myogenesis.

In muscle pathology and ageing contexts, where there is a predominant switch of the fiber phenotype from fast to slow [32,33], IGF2 was also able to orchestrate the development of fast myofibers by acting as a twitch motor unit during secondary myogenesis. The modulation of IGF2 expression had a dramatic impact on the amount of fast myofibers in the respiratory (intercostal and diaphragmatic) muscles, likely lessening cardio-respiratory dysfunction related to DMD. IGF2 targeting was suggested as a feasible therapeutic strategy, since IGF2 has a small size and consequently it could be easily distributed to a skeletal muscle target [34]. However, the IGF2R signaling pathways involved in muscle repair and disease remain to be identified.

## 2. Structure, Genomic Organization and Gene Imprinting of IGF2R

Imprinting genes are those whose expression is determined by one’s parents. They occur in discrete clusters that are regulated by DNA elements called imprinting control regions (ICR). The two copies of one imprinted gene are characterized by methylation of cytosine–guanine base pairs. This modification originates in the paternal germ cells—after adding a methyl group, the chromatin becomes inaccessible to transcription machinery, so the gene is silenced. The IGF2/H19 locus is one of the imprinted gene clusters in human chromosome 11p15.5 or mouse distal chromosome 7 and plays a primary role in muscle cell development [35]. The expression of this cluster is regulated by a distant enhancer downstream of the H19 coding region. A recent study presents paxillin (PXN), a focal adhesion protein, as a transcriptional regulator of the IGF2 and H19 genes; in particular, it has the opposite effect on the activity of the IGF2 and H19 promoters [36]. The knockdown of PXN in human HepG2 cells allows for an increase in the activity of the H19 promoter and at the same time a decrease in the activity of the IGF2 promoter [36]. In a recent study, it was demonstrated that the loss of imprinting (LOI) in a mouse model of Beckwith–Wiedemann syndrome (BWS) results in impaired muscle differentiation and hypertrophy. It was also proposed that there is a signaling pathway in which IGF2 overexpression allows for an overactivation of mitogen-activated protein kinase (MAPK) signaling, while a loss of H19 long non-coding RNA (lncRNA) prevents the regulation of p53 levels, resulting in reduced AKT/mTOR signaling [35].

The *IGF2R* is an example of differential imprinting in the human and mouse genomes; the *IGF2R* is repressed on the paternal chromosome in the murine genome, but the same gene is expressed from both alleles in humans. For humans, the study was conducted on several tissues—adult liver, placenta, fetus, kidney, adrenal, brain, intestine, heart, tongue, skin and muscle: in all these samples both IGF2R alleles were expressed more or less at the same level. Accordingly, it was established that this character is subject to Mendelian segregation, escaping imprinting. As a plausible explanation for this phenomenon, Kalscheuer et al. suggested that the stages of initiation and maintenance of imprinting could be under the control of trans-acting factors that could act differently in mice compared to in humans [37]. They also hypothesized an alternative explanation based on the structural difference of the mouse and human *IGF2R* genes, as an “imprinting box” was previously discovered that could be modified by methylation in the female gamete and allowed maternal expression [37]. The *IGF2R* gene is located on mouse chromosome 17: it is composed of 48 exons and encoded for a 2482-amino acid protein. Exons 1–46 comprise the extracellular part of the receptor, while the transmembrane portion and the cytoplasmic region are located, respectively, on exon 46 and 46–48 [38].

## 3. IGF2R-Dependent Pathway

M6P/IGF2-R lacks intrinsic kinase activity, and the role of G-proteins in its downstream pathway has been investigated. Functionally, G-proteins are a class of proteins that interact with multi-spanning receptors (seven transmembrane receptors or heptahelical receptors). Some studies have speculated that M6P/IGF2-R, although characterized by a single-spanning structure, might initiate signaling cascades through G-proteins in a direct or indirect manner. It is well known that the pertussis toxin exerts its activity by binding and blocking the activation of the α subunit of the Gq/11-protein [39]. El-Shewy et al. showed that the use of this toxin is able to inhibit the function of M6P/IGF2-R, therefore suggesting the involvement of G-proteins in the downstream pathway of M6P/IGF2-R. Pre-treatment of Human embryonic kidney 293 cells (HEK-293) cells with the pertussis toxin can significantly reduce the level of extracellular signal-regulated kinase 1/2 (ERK1/2) phosphorylation resulting from the interaction of IGFs with their receptors. The indirect activity of these receptors is carried on through parallel activation of G-protein-coupled receptors (GPCR). They also observed that the administration of IGF1 and IGF2 ligands activates sphingosine kinase (SK) 4, which is translocated from the cytosol to the plasma membrane. There, it promotes an increase in extra- and intracytoplasmic levels of sphingosine-1-phosphate (S1P). S1P’s interaction with its G-protein-coupled receptor represents a general mechanism for indirect G-protein-dependent signaling of M6P/IGF2-R resulting in ERK1/2 phosphorylation [40]. Conversely, the direct activity of IGF2R and G-proteins was hypothesized by Nishimoto et al. [41,42]: based on their observations, a region of the cytoplasmatic domain of M6P/IGF2-R may contain aminoacidic residues (2410–2423) that directly bind and activate G-proteins, in particular, Gi-2. This is supported by evidence that the use of both human and rat antibodies targeting aminoacidic residues is able to inhibit the activation of Gi-2 resulting from M6P/IGF2-R stimulation.

## 4. Functions of IGF2R

### 4.1. IGF2R Expression Levels Regulate Cardiac Development and Remodeling

The expression of the *IGF2R* gene is particularly abundant in embryo hearts. IGF2R-deficient mice display dramatic cardiac dysfunction and heart failure development. In adults, low levels of IGF2R expression lead to heart disease and apoptosis in cardiac myocytes [43], while upregulation determines myocardial infarction, remodeling [44] and hypertrophy [45]. In particular, the IGF2R activates phospholipase C (PLC) through the heterotrimeric G-protein-coupled receptor: this interaction is mediated by αq G subunits (Gαq) that in turn allow the function of different enzymes such as the protein kinase C-α (PKC-α), Ca^2+^-calmodulin-dependent protein kinase II (CaMKII) and ERK kinase—all upregulated in cardiac hypertrophy [46]. Alternatively, the modulation of IGF2R can enhance cardiomyocyte apoptosis and cardiac contractility by inhibiting protein kinase A (PKA) phosphorylation [47]. A recent study showed that IGF2R expression is negatively controlled by the cardioprotective heat shock transcription factor 1 (HSF1). Antitumor drugs such as doxorubicin (DOX), meanwhile, lead to high levels of IGF2R expression in cardiomyocytes, through a decrease in HSF1, and trigger cardiac apoptotic processes [48]. IGF2R expression in the heart may also mediate increased sizes of cardiomyocytes, in a manner dependent on PKA activation, and mediate atrial natriuretic peptide (ANP), calcium-dependent channels (SERCA) and phospho-troponin I signaling, as described recently by Wang et al. [49].

### 4.2. IGF2R Modulates Vascular Remodeling and Skeletal Muscle Growth

IGF proteins play an essential role in skeletal muscle homeostasis and vascular mechanisms involving smooth muscle cells (SMCs). The latter are driven by IGF2 in the development and migration processes occurring during vascular growth or in response to vascular damage. A study has verified the role of IGF2 in SMC migration by studying the interaction between the cellular repressor of E1A-stimulated gene (CREG) factor and M6P/IGF2-R. Specifically, when the CREG factor binds M6P/IGF2-R, it is able to inhibit the SMC proliferation process and the migration process. Further studies showed that CREG knockdown leads to an increased release of IGF2, mitigating its internalization and partly restoring the migration process of the SMCs. Accordingly, the use of an anti-human IGF2-neutralizing antibody on a CREG knockdown population promotes the inhibition (in a concentration-dependent manner) of the SMCs’ migration process [50]. Despite the shortage of detailed articles, an indirect point of view of the solid connection between vascular remodeling and IGF2R is offered by Ca^2+^ signaling. A comprehensive body of literature has already demonstrated that IGF2R triggers several intracellular signaling pathways aimed at Ca^2+^ mobilization [41]. This cascade may occur through an increase in PKC-α phosphorylation or in a Gαq-dependent manner [51], such as for controlling hypertrophy in cardiac cells, or through PLC-induced interactions between IGF2R and G(i) protein, as in endothelial progenitor cells (EPCs) [52]. In the latter case, the upstream role of IGF2R possibly affects the migration, adhesion and invasion of EPCs in the neovascular zone, therefore raising the importance of IGF2R for vessel network formation, both in embryonic and in post-ischemic vasculo-genesis. Vascular SMCs, composing the medial layer of blood vessels, are also essential for the maintenance of post-natal vascular homeostasis, and their correct functionality is subordinated to Ca^2+^ signaling. Intracellular calcium is likely to regulate the mechanical properties of SMCs through a tight modulation of α5β1 integrin, α-SMA and cell–ECM interactions. The Ca^2+^ dynamic across cells may activate the elasticity and the adhesion properties of vascular SMCs, with physiologically important consequences on vascular tone and resistance, and blood flow and pressure [53].

A more complex signaling pathway underlying Ca^2+^ entry and exit from cells [54] is tuned by ATP-dependent pumps, which counterbalance the calcium ion levels and the electrolyte homeostasis. Among these pumps, sarcoplasmic/endoplasmic reticulum (SR) Ca^2+^ATPase (SERCA) is the one in charge of the Ca^2+^ homeostasis within the reticulum, with a role susceptible to the type of cells [55,56]. There are three isoforms of SERCA characterized by tissue-specific expression. Briefly, the fast twitch skeletal muscle isoform SERCA1 has been found in the heart, and, to a lesser extent, in the liver, kidney, brain and pancreas [57]. Variants of SERCA2 have been detected preferentially in cardiac, skeletal and vascular smooth muscle [58]. SERCA3 isoforms are heterogeneously expressed through tissues and, conversely to the others, are characterized by a low affinity to Ca^2+^ [59]. Altered levels of SERCA proteins lead to aberrant calcium flux dynamics, which are responsible for the reduced contractility and dysfunction of SMCs observed in numerous diseases including cardiomyopathies, atherosclerosis, metabolic syndromes, and diabetes [60,61]. Although this evidence implies a connection between SERCA, IGF2R and calcium flux, a thorough explanation of the causal relationships is yet to be provided. Recently, we have identified a possible pathway in the context of muscular dystrophies, which are often associated with Ca^2+^ dysfunction: the contractile function of muscle fibers is dependent on the expression of many proteins involved in the calcium cycle between the cytosol and SR. Ca^2+^ signaling includes the ryanodine receptor, which is the SR Ca^2+^ release channel; the troponin protein complex, which leads to contraction; the extracellular Ca^2+^ reuptake pump, which mediates the flux of Ca^2+^ into the SR by a mechanism called store-operated Ca^2+^ entry (SOCE); and calsequestrin, the Ca^2+^ storage protein in the SR. In addition, several Ca^2+^-binding proteins are present in muscle tissue such as calmodulin, annexins, myosin, calcineurin and calpain [62]. In our study, we found that IGF2R expression is increased in dystrophic muscles, and IGF2R and the store-operated Ca^2+^ channel CD20 share a common hydrophobic binding motif stabilizing their association. We verified that the intravenous administration of an anti-IGF2R-neutralizing antibody facilitates IGF2/IGF1R interactions, while the occurrence of CD20 phosphorylation activation promotes the entrance of Ca^2+^ ions into the sarcoplasm [63]. Based on this evidence, we proposed a signaling pathway to explain the activation of SERCA and the Ca^2+^ flux through cells. Among the pathway proteins, STIM1 and ORAI1 are engaged in SOCE regulation and activation. STIM1 acts as a sensor of Ca^2+^ concentration in cellular stores and undergoes a horizontal movement in the SR membrane when the ER is calcium-depleted. Due to this shifting, STIM1 interacts with the membrane channel protein ORAI1 and causes calcium to enter the cell. After the replenishment of calcium stores, the ORAI1/STIM1 interaction dissolves and the Ca^2+^ influx stops. CD20 phosphorylation decreases the interaction between CD20 and ORAI1 in store-depleted myoblasts, largely in anti-IGF2R-treated myoblasts.

In dystrophic muscle, SERCA activity is reduced [64,65] leading to higher cytoplasmic levels of calcium ions. After anti-IGF2R treatment, calcium uptake is activated by CaMKII-dependent regulation of SERCA1: the blockade of IGF2R allows the activation of calcineurin, which dephosphorylates the nuclear factor of activated T cells (NFAT), which, consequently, shuttles into the nucleus, promoting activation of the genes involved in myogenic differentiation. Moreover, the binding of anti-IGF2R to domain 11 of IGF2R activates IGF2R/Gαi2 interactions, preventing the interplay with IGF2. This event increases the bioavailability of IGF2 for IGF1R and promotes the activation of PI3-K/AKT/mTOR signaling involved in expression of myogenic genes. Our results demonstrated that the blockade of IGF2R in mdx muscles rescued the murine dystrophic phenotype and increased force production. The in vivo experiments also revealed a marked vascular remodeling consisting of structural modifications resulting in higher linearization and wrapping of muscle fibers. It was conceivable that EPCs and pericyte cells accounted for the amelioration of the microvasculature and the blood supply. Adhesion and migration of EPCs could be affected directly by the IGF2R blockade, while pericytes cells, which present a contractile activity, could sense the calcium uptake activation [66]. Initially, pericytes had been discovered as mural cells able to provide capillary stability by interacting with endothelial cells. This classification was exceeded by anatomical and morphological evidence [67] demonstrating that pericytes could not only have a contractile activity, but they could regulate blood flow, capillary diameters and vascular tone [66,68,69]. In turn, pericytes’ behavior in microcirculation can be regulated by upstream and downstream signaling, depending on the tissues and cell types. For instance, in healthy conditions, pericytes’ coverage of the retina is essential for maintaining the contact with the endothelium and the integrity of the vascular barrier. Diabetic mice exhibit altered retinal vasal permeability caused by high levels of macrophage-secreted cathepsin D (CD). CD has been shown to disrupt the pericyte–endothelial junction either by increasing Rho/ROCK-dependent cell contractility [70] or by binding to IGF2R via 2M6P binding sites and changing PKC-α–CaMKII signaling [71].

Additional demonstrations of the association of IGF2R with insulin resistance and glucose homeostasis are based on the discovery of IGF2R genetic variants and soluble circulating IGF2R [72] in both type 1 (T1DM) and type 2 (T2DM) diabetes mellitus [73,74]. In T2DM, hyperglycemia seems to severely affect the islet capillary pericytes in terms of reduced numbers, improper islet coverage, altered calcium flux sensitivity, and relaxation phenotype shift [75]. As a response, diabetic capillaries dilate, blood pressure increases, and islets lose the ability to adapt and control their blood flow. Finally, high glucose concentrations and streptozotocin-induced diabetic conditions lead to abnormal activation of the IGF2R pathway and a downstream signaling for the expression of proteins related to hypertrophy in cardiac tissues, and to apoptosis in cardiomyocytes [76].

Taken together, this evidence suggests the importance of IGF2R in the pathogenesis or treatment of disorders related to energy metabolism, vascular remodeling and muscle homeostasis. However, unraveling the signaling of the whole process within different tissues requires further and extensive investigation.

### 4.3. IGF2R Is Involved in Carcinogenesis

**Figure 1:**
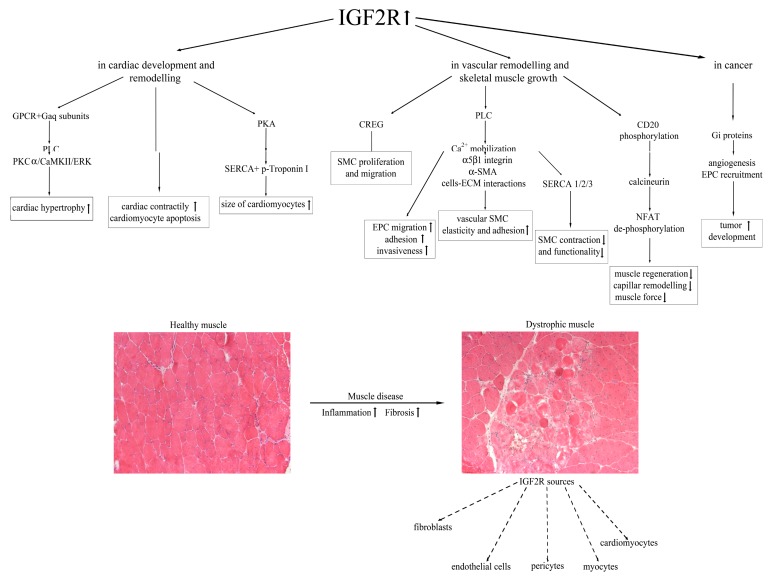
A schematic model of the IGF2R-dependent mechanisms leading to cardiac and skeletal muscle impairment, and cell sources of IGF2R expression in dystrophic muscle.

As described above, the proteins that constitute the IGF system—IGF1/IGF2, the surface receptors, and the IGF-binding proteins—regulate a plethora of functions related to growth and development operating through AKT1, mitogen-activated protein kinase (MAPK) and the phosphatidylinositol 3-kinase (PI3K) [77]. Consequently, dysfunction in this complex system is often associated with cancer. The upregulation of IGF2 was described in colorectal cancers (CRC) due to epigenetic mechanisms, and it was associated with poor survival [78]. In particular, IGF2 was dramatically overexpressed in tumorigenic clones related to IGF1, leading to constitutive activation of IGF1R and AKT [79] and to malignant modulation of apoptosis and stemness [80]. In addition, the loss of IGF2 imprinting was associated with esophageal adenocarcinoma [81], while the hypomethylation of one of the IGF2 promoters caused dysfunction in the transcriptional regulator Kruppel-like factor 4 (KLF4) in humane prostate cancer [82]. Genetic mutations in IGF2R can modify the bioavailability of IGF2 so that cancer cells can dramatically proliferate. Different studies demonstrated that IGF2R expression could be involved in the development of hepatocarcinoma, breast and ovarian human cancers by encoding for a tumor suppressor gene [83]. In particular, the loss of heterozygosity (LOH) at the M6P/IGF2R gene locus on 6q26–27 chromosome seemed to be associated with the invasiveness of breast cancers, while M6P/IGF2R point mutations were identified in hepatoma, gastrointestinal (mainly associated with microsatellite instability) and prostate tumors [84]. This condition likely led to uncontrolled IGF2 upregulation that enhanced cancer growth and survival by binding to IGF1R. In particular, the work of Delaine et al. [85] showed a new hydrophobic patch on the domain 11 of the IGF2R that is fundamental for the high binding affinity of IGF2/IGF2R. The first direct demonstration of IGF2R in tumor growth came from the work of Chen et al., in which they described how the downregulation of M6P/IGF2R expression in adenocarcinoma cells led to increased cell proliferation and decreased susceptibility to apoptosis, according to the bioavailability of TNFα and activated TGF-β. In addition, this condition was probably hampered by the action of IGF2 and cathepsins B and D [86]. Interestingly, ligands other than IGF2 can bind to IGF2R: among them are urokinase-type plasminogen activator receptor (uPAR) and retinoic acid, whose activity allows the internalization of IGF2 [7]. All the IGF2R-dependent pathways are summarized in Figure 1.

## 5. Conclusions

Fetal growth and post-natal growth are closely regulated by the insulin-like growth factor axis: alterations in the IGF signaling pathways could cause severe dysfunction in somatic growth and development and be responsible for tumor proliferation. We have recently demonstrated in mdx mice that intravenous administration of an anti-IGF2R neutralizing antibody significantly upregulated muscle regeneration and decreased fibrosis, leading to the rescue of the pathological phenotype. The inhibition of IGF2R resulted in an increase in intracellular Ca^2+^ in myoblasts and increased SERCA1 activity, possibly operating through CD20. This condition allowed NFAT dephosphorylation and its translocation into the nucleus. The dystrophic phenotype rescue activated in vivo by the anti-IGF2R antibody was further corroborated by higher numbers of structurally more linear microvessels enveloping myofibers. It is likely that anti-IGF2R acts on pericyte function, determining normalization of the vascular wall and consequent amelioration of the oxygenation of the dystrophic muscle. As contractile cells, pericytes can regulate their tone and contraction depending on the intracellular calcium concentration and consequently tune the capillary diameter and blood flow [69].

This is an exciting time in our understanding of muscular dystrophies. Increasing knowledge of IGF2R’s role in muscle disease is starting to suggest new therapeutic approaches. A challenge for the future will be to understand how IGF2R interacts with other components of the muscle system to influence muscular dystrophy progression. Similarly, genetic and epigenetic changes affecting *IGF2R* need to be considered. Therefore, targeting IGF2R may be a potential therapeutic strategy for muscular dystrophies.

## Abbreviations

*α*-SMA	alpha smooth muscle actin
ANP	atrial natriuretic peptide
CaMKII	Ca^2+–^calmodulin-dependent protein kinase II
CREG	Cellular Repressor of E1A-stimulated Gene
ECM	extracellular matrix
EPC	endothelial progenitor cell
Gαq	αq G subunits
GPCR	G-protein-coupled receptors
NFAT	nuclear factor of activated T cells
PKA	protein kinase A
PKC-α	protein kinase C-α
PLC	phospholipase C
SERCA	sarcoplasmic/endoplasmic reticulum (SR) Ca^2+^ATPase
SMC	smooth muscle cell
